# Ultrahigh pressure phase stability of AlB_2_-type and CaC_2_-type structures with respect to Fe_2_P-type and Ni_2_In-type structures of zirconia

**DOI:** 10.1038/s41598-023-44508-6

**Published:** 2023-10-13

**Authors:** Yahya Al-Khatatbeh, Khaldoun Tarawneh

**Affiliations:** Department of Basic Sciences, Princess Sumaya University for Technology, Amman, 11941 Jordan

**Keywords:** Phase transitions and critical phenomena, Structure of solids and liquids

## Abstract

Using density-functional theory, we have performed *first-principles* calculations to test the phase stability of the hexagonal AlB_2_-type and tetragonal CaC_2_-type phases at ultrahigh pressures with respect to the experimentally observed hexagonal Fe_2_P-type phase and the recently predicted (as post-Fe_2_P) hexagonal Ni_2_In-type phase of ZrO_2_. The phase relations among the four phases have been thoroughly investigated to better understand the high-pressure behavior of ZrO_2_, especially the upper part of the pressure phase transition sequence. Our enthalpy calculations revealed that the transformation from Ni_2_In phase to either AlB_2_ phase or CaC_2_ phase is unlikely to happen. On the other hand, a direct phase transition from Fe_2_P phase to Ni_2_In, CaC_2_ and AlB_2_ phases is predicted to occur at 325 GPa, 505 GPa and 1093 GPa, respectively. A deep discussion has been made on the Fe_2_P → Ni_2_In and Fe_2_P → CaC_2_ transitions in terms of the volume change, the coordination number (CN) change, and the band gap change to obtain a better prediction of the favored post-Fe_2_P phase of ZrO_2_. Additionally, the equation of state (EOS) parameters for each phase have been computed using Birch-Murnaghan EOS. To further investigate the phase stability testing, we have studied the components of the enthalpy difference to explore their effect on our findings, and found that all predicted transitions from Fe_2_P phase are driven by the volume reduction effect when compared to the slight effect of the electronic energy gain.

## Introduction

Zirconia (ZrO_2_) is a transition-metal dioxide that is involved in a wide range of interesting applications^1–11^. The use of ZrO_2_ covers many applications such as coatings, photocatalysis, and gate insulators^1–11^. In the last few decades, investigating the high-pressure behavior of ZrO_2_ has attracted great attention both experimentally and theoretically^12–29^. This attention has been given to zirconia as a result of its interesting properties like structural, mechanical, and optical properties. Therefore, studying the high-pressure behavior of such dioxide and other oxides can be considered as an important research field via both experimental measurements and theoretical calculations^12–38^.

**Figure 1:**
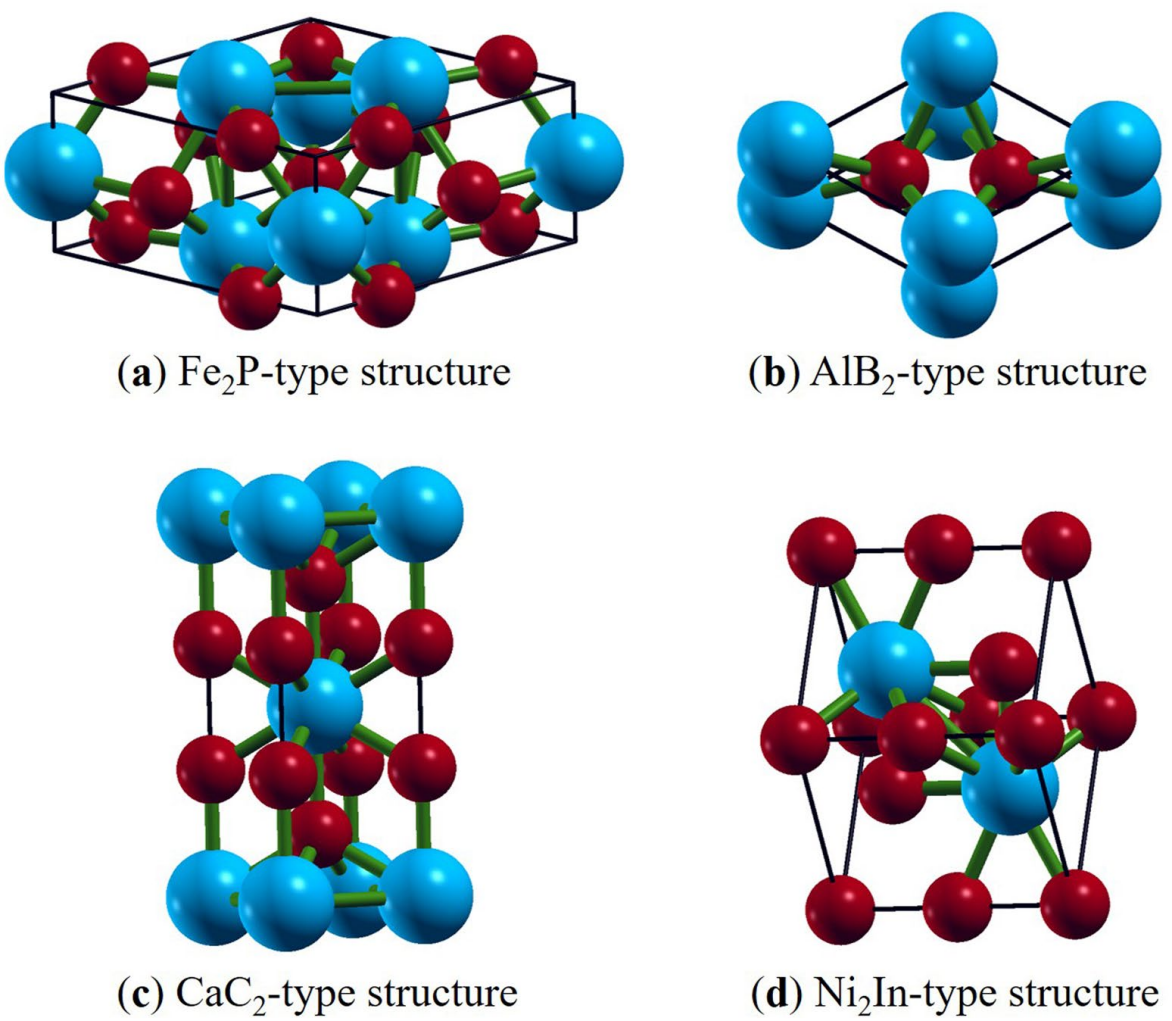
Crystal structures of ZrO_2_ phases (generated using XCrySDen software^45^). The blue spheres represent the Zr atom, while the red spheres represent the O atom. (**a**) Fe_2_P-type: Crystal structure: Hexagonal, space group: $P\overline{6}2m$. (**b**) AlB_2_-type: Crystal structure: Hexagonal, space group: *P*6/*mmm*. (**c**) CaC_2_-type: Crystal structure: Tetragonal, space group: *I4*/*mmm*. (**d**) Ni_2_In-type: Crystal structure: Hexagonal, space group: *P*6_3_/*mmc*.

A great research effort, using experimental and theoretical work^12–29^, has been made to have a better knowledge of the high-pressure/temperature behavior and phase stability of different ZrO_2_ phases (Fig. 1), with fairly comparable results between measurements and calculations^12–29^. The observed (and predicted) high-pressure phase transition sequence in zirconia is as follows: Monoclinic baddeleyite → orthorhombic OI → orthorhombic OII → hexagonal Fe_2_P^13,23–25,27,39,40^. Therefore, up to the maximum pressures achieved, Fe_2_P is the only phase experimentally observed as a post-OII phase for zirconia^23^ in agreement with *first-principles* calculations^20–23^. In this regard, the experimental observations using diamond-anvil cell (DAC) have shown that Fe_2_P-ZrO_2_ becomes stable at extreme pressure–temperature (*P*–*T*) conditions (175 GPa, 3000 K)^23^, while the theoretical predictions have shown that its stability begins at lower pressures of 94–143 GPa^20–23^.

Additionally, recent calculations have predicted Ni_2_In-type structure to be the highest-pressure phase of ZrO_2_ at megabar pressures^21,22^, where Fe_2_P → Ni_2_In transition is predicted to take place at 317–370 GPa. However, it has been predicted that CaC_2_-type structure is the most stable phase for the similar dioxide TiO_2_, where the calculated transition pressure of Fe_2_P → CaC_2_ is found to be 647–689 GPa^41,42^. Moreover, it has been reported that the Ni_2_In phase is predicted to transform to another hexagonal AlB_2_-type structure in strontium hydride (SrH_2_)^43^ for which this hydride seems to share some similarities with the transformation sequence of zirconia.

It is important to note that although a reentrant transition of OII has been predicted recently for ZrO_2_^44^, we test the transition from Fe_2_P phase rather than OII to better compare our findings with previous reports on the transition pressures with respect to Fe_2_P phase. Additionally, Fe_2_P-ZrO_2_ is the highest-pressure phase experimentally observed until now^23^, which supports our consideration for the high-pressure phase stability to be with respect to Fe_2_P phase. However, we should note that the transition pressures from Fe_2_P or OII to any new high-pressure phase (in particular, OII → Ni_2_In and Fe_2_P → Ni_2_In transitions) are similar^22^ due to the slight change in enthalpy between Fe_2_P and OII^35,44^. Consequently, our calculations are performed to explore the relative stability of other phases with respect to Fe_2_P phase rather than OII phase.

In this work, we use DFT calculations to investigate the stability of CaC_2_ and AlB_2_ phases with respect to Fe_2_P and Ni_2_In phases at high pressures (Fig. 1). In detail, we test the stability of CaC_2_ and AlB_2_ phases with respect to Ni_2_In phase as possible post-Ni_2_In phases. Then, we explore the phase stability of the latter three phases with respect to Fe_2_P, especially the Fe_2_P → Ni_2_In and Fe_2_P → CaC_2_ transitions to highlight the similarities and/or differences between ZrO_2_ and the similar dioxide TiO_2_. In other words, we aim to test whether ZrO_2_ follows the same high-pressure sequence of TiO_2_ at extreme pressures or not, given that both dioxides share a proven similar transition sequence up to Fe_2_P phase while DFT studies have reported the post-Fe_2_P to be Ni_2_In for ZrO_2_^21,22^ and CaC_2_ for TiO_2_^41,42^. As a result, this investigation will hopefully yield a better understanding of the behavior of such dioxides at ultrahigh pressures.

### Computational details

In order to investigate the phase stability and the equations of state (EOSs) of all tested phases of ZrO_2_, we used static *first-principles* computations performed within the framework of density-functional theory (DFT)^46^. The projector-augmented wave (PAW) formalism^47,48^ was used to treat the interactions between the zirconium (Zr) and oxygen (O) atoms with the valence configuration of 4*s*^2^4*p*^6^5*s*^2^4*d*^2^ for Zr, and 2*s*^2^2*p*^4^ for O. Following previous theoretical high-pressure studies carried out on ZrO_2_, HfO_2_ and TiO_2_^21,25,32–34^, the electronic exchange and correlation effects were treated within the generalized gradient approximation (GGA)^49^. We performed our calculations using the Quantum ESPRESSO package^50^ with an energy cutoff of 80 Ry and Γ-centered *k*-point meshes^51^. Our calculations yielded sufficient convergence to better than 10^–5^ Ry in the total energies for all phases and pressures were converged to better than 0.1 GPa. The Brillouin zone integration was performed using the following *k*-point meshes for the ZrO_2_ phases: 8 × 8 × 12 for Fe_2_P, 20 × 20 × 16 for Ni_2_In, 15 × 15 × 15 for AlB_2_, and 12 × 12 × 8 for CaC_2_. For a fixed volume, all internal degrees of freedom and unit-cell parameters of the structure were optimized simultaneously during the geometry optimizations. The ground-state energy for each phase was determined for 14–17 volumes, and the EOS parameters for each phase were obtained by fitting the total energy as a function of volume to a second-order Birch-Murnaghan equation of state (BM-EOS)^52^ (Table 1).

**Table 1:** Calculated equations of state for Fe_2_P, Ni_2_In, CaC_2_ and AlB_2_ phases of ZrO_2_. Our EOS is determined from GGA calculations using the second-order BM-EOS^52^. For comparison, we list other calculated EOSs. 1σ uncertainties are given in parentheses.

Phase	Equation of state			Reference
	*V*_0_ (Å^3^)	*K*_0_ (GPa)	*K*_0_’	
Fe_2_P	30.34	272	4 (fixed)	Ref.^23^
	30.43	260	4.18	Ref.^23^
	30.17	248	3.76	Ref.^22^
	30.94 (0.03)	272 (2)	4 (fixed)	Ref.^21^
	30.98	255	4.38	Ref.^20^
	30.40 (0.02)	274 (1)	4 (fixed)	This work
Ni_2_In	29.21	239	3.86	Ref.^22^
	31.81 (0.13)	200 (5)	4 (fixed)	Ref.^21^
	31.27 (0.14)	201 (5)	4 (fixed)	This work
CaC_2_	29.78 (0.02)	255 (1)	4 (fixed)	This work
AlB_2_	30.50 (0.03)	227 (1)	4 (fixed)	This work

## Results and discussion

### Compressibility and equation of state parameters

**Figure 2:**
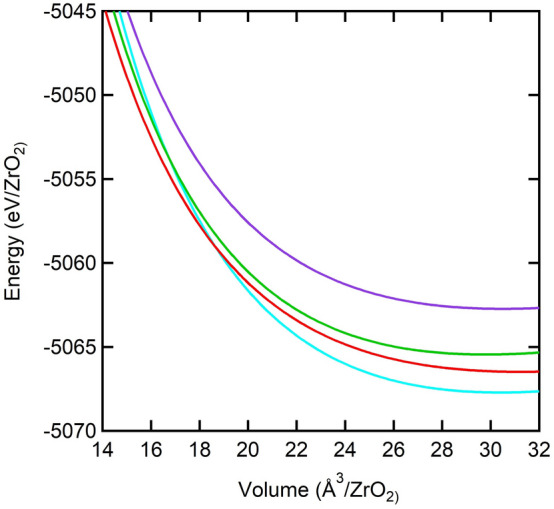
Electronic energy versus volume of one formula unit for ZrO_2_ phases (Fe_2_P: blue, Ni_2_In: red, CaC_2_: green, AlB_2_: purple) as determined by GGA calculations using BM-EOS ^52^.

To obtain the equation of state parameters, we have fit our energy-vs-volume data to BM-EOS^52^ (Fig. 2). The third-order forms of BM-EOS for the pressure (*P*) as a function of volume (*V*) and energy (*E*) as a function of volume, respectively, are:

$$P(V) = \frac{3}{2}K_{0} \left[ {\left( {\frac{V}{{V_{0} }}} \right)^{{ - \frac{7}{3}}} - \left( {\frac{V}{{V_{0} }}} \right)^{{ - \frac{7}{3}}} } \right]\left[ {1 + \frac{3}{4}\left( {\left( {K^{\prime}_{0} - 4} \right)\left( {\left( {\frac{V}{{V_{0} }}} \right)^{{ - \frac{2}{3}}} - 1} \right)} \right)} \right]$$

$$E(V) = \frac{{9K_{0} V_{0} }}{2}\left[ {\frac{1}{2}\left( {\left( {\frac{V}{{V_{0} }}} \right)^{{ - \frac{2}{3}}} - 1} \right)^{2} } \right]\left[ {1 + \left( {K^{\prime}_{0} - 4} \right)\left( {\frac{1}{2}\left( {\left( {\frac{V}{{V_{0} }}} \right)^{{ - \frac{2}{3}}} - 1} \right)} \right)} \right] + E_{0}$$

where *V*_0_ is the volume at zero pressure, *K*_0_ is the zero-pressure bulk modulus, *K*_0_’ is the first pressure derivative of the bulk modulus at zero pressure, and *E*_0_ is the total energy at zero pressure.

The latter equations will be reduced to the second-order forms below when *K*_0_’ = 4:

$$P(V) = \frac{3}{2}K_{0} \left[ {\left( {\frac{V}{{V_{0} }}} \right)^{{ - \frac{7}{3}}} - \left( {\frac{V}{{V_{0} }}} \right)^{{ - \frac{7}{3}}} } \right]$$

$$E(V) = \frac{{9K_{0} V_{0} }}{4}\left( {\left( {\frac{V}{{V_{0} }}} \right)^{{ - \frac{2}{3}}} - 1} \right)^{2} + E_{0}$$

In this section, we investigate the compressibility of the proposed phases, and therefore we use the second-order *E*(*V*) BM-EOS to calculate the equation of state parameters for each phase with a particular focus on the bulk modulus.

In Table 1, we list our computed equation of state parameters for each phase of ZrO_2_ as well as any other previously reported values^20–23^ for comparison purposes, where a good agreement can be noted. However, this agreement is less pronounced for the Ni_2_In case for which one previous study^22^ has reported an ~ 19% larger bulk modulus compared to ours, but with smaller *K*_0_’ and *V*_0_, which are inversely proportional to *K*_0_, making this difference (although not unreasonable) explainable. To our knowledge, CaC_2_ and AlB_2_ phases have not been tested previously for ZrO_2_, so there are no reported values to compare with.

**Figure 3:**
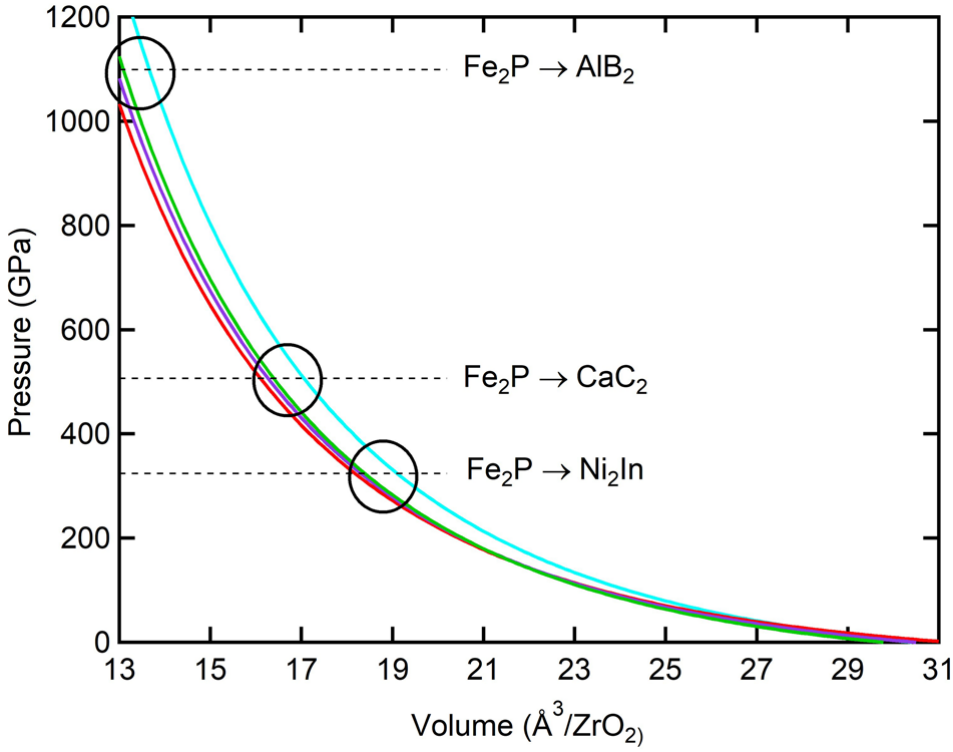
Pressure versus volume of one formula unit for ZrO_2_ phases (Fe_2_P: blue, Ni_2_In: red, CaC_2_: green, AlB_2_: purple) as determined by GGA calculations using BM-EOS^52^. The horizontal dashed lines represent the calculated transition pressures from Fe_2_P phase. The open circles show the large volume change across transitions from Fe_2_P to Ni_2_In (bottom circle), CaC_2_ (middle circle), and AlB_2_ (top circle) phases.

From Table 1, it can be concluded that our calculations show that Fe_2_P is the least compressible phase and Ni_2_In is the most compressible one, while CaC_2_ and AlB_2_ have intermediate compressibilities. In detail, the order of incompressibility from lowest to highest is as follows: Ni_2_In → AlB_2_ → CaC_2_ → Fe_2_P. Additionally, considering the transitions from the Fe_2_P phase (see next section), we note that the values of the bulk modulus decrease associated with Fe_2_P → Ni_2_In, Fe_2_P → CaC_2_ and Fe_2_P → AlB_2_ transitions are ~ 27%, 7% and 17%, respectively. Compared to the other two transitions, the bulk modulus change is more reasonable when Fe_2_P transforms to CaC_2_ phase since we do not expect a significant change for transitions to higher-pressure phases in ZrO_2_^21–25^ and similar dioxides^32,33^. Moreover, we also note that the Fe_2_P → CaC_2_ transition is associated with an increase in density (*V*_0_: 30.40 Å^3^ → 29.78 Å^3^) in contrast to the Fe_2_P → Ni_2_In and Fe_2_P → AlB_2_ transitions that are related to a decrease in the density of Ni_2_In and AlB_2_ phases (Fig. 3, Table 1).

### Phase stability and phase transitions

**Figure 4:**
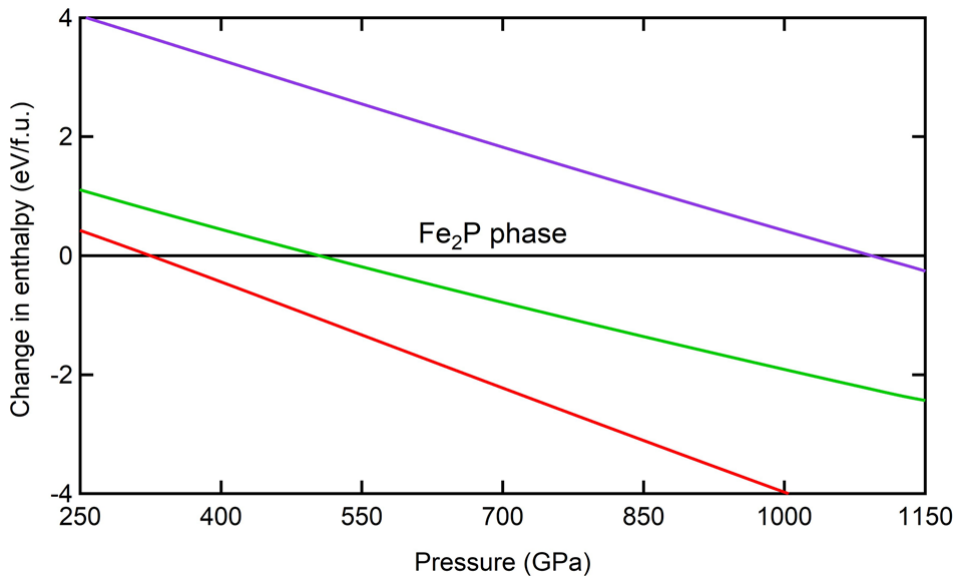
Enthalpy difference with respect to Fe_2_P phase versus pressure of one formula unit as determined by GGA calculations for ZrO_2_. The transition pressures across transitions from Fe_2_P to Ni_2_In (red), CaC_2_(green) and AlB_2_ (purple) phases are shown. The transition from Ni_2_In to either CaC_2_ or AlB_2_ is unlikely to occur.

Our enthalpy calculations (Fig. 4, Table 2) show that the transition from Ni_2_In to either CaC_2_ or AlB_2_ is not likely. Eliminating the possibility of Ni_2_In → AlB_2_ transition leads to the conclusion that SrH_2_^43^ behaves differently at ultrahigh pressures compared to ZrO_2_. Although our predictions indicate that both CaC_2_ and AlB_2_ phases are not stable with respect to Ni_2_In, a special attention has been given to the previously predicted CaC_2_-type structure of the similar transition-metal dioxide TiO_2_. In detail, since the Ni_2_In → CaC_2_ transition is not possible in ZrO_2_, one should conclude that Fe_2_P phase should transform to either Ni_2_In (previously predicted for ZrO_2_^21,22^ and HfO_2_^34^) or CaC_2_ (previously predicted for TiO_2_^41,42^) (Fig. 4, Table 2).

**Table 2:** Calculated transition pressures across Fe_2_P → Ni_2_In, Fe_2_P → CaC_2_ and Fe_2_P → AlB_2_ phase transitions in ZrO_2_. For comparison, we list other calculated results.

Phase transition	Transition pressure (GPa)	Reference
Fe_2_P → Ni_2_In	370 GPa	Ref.^22^
	311–317 GPa	Ref.^21^
	325 GPa	This work
Fe_2_P → CaC_2_	505 GPa	This work
Fe_2_P → AlB_2_	1093 GPa	This work

However, the enthalpy difference versus pressure curves (Fig. 4, Table 2) show that the Fe_2_P → Ni_2_In transition pressure is 325 GPa that fairly agrees with previous reports^21,22^, while the Fe_2_P → CaC_2_ transition occurs at a higher pressure of 505 GPa. On the other hand, the transition of Fe_2_P → AlB_2_ is predicted at a very extreme pressure of 1093 GPa that exceeds the possible experimental pressure range, which adds one more reason why to focus on Fe_2_P → Ni_2_In versus Fe_2_P → CaC_2_ transitions. Therefore, for the sake of better prediction of the favorable transition from Fe_2_P phase, we further investigate the volume change, the CN change and the band gap change across Fe_2_P → Ni_2_In and Fe_2_P → CaC_2_ transitions.

### Volume reduction and coordination number change across phase transitions

The pressure–volume curves of ZrO_2_ phases show a large predicted volume change across both Fe_2_P → Ni_2_In and Fe_2_P → CaC_2_ transitions of 4.7% and 4.1%, respectively (Fig. 3, Table 3), where this is consistent with the trend of compressibility change across these transitions. Additionally, our analysis for the effect of the components of enthalpy difference has shown that both transitions are much more driven by the volume reduction term compared to the electronic energy gain (Table 3). Although the effect of the electronic energy gain is predicted to be small in all transitions from Fe_2_P, it has been found to be very minor for the Fe_2_P → CaC_2_ transition (Table 3). However, these large differences in enthalpy across transitions (Table 3) are not unexpected as both transitions require increasing of the CN; from 9 to 11 for Fe_2_P → Ni_2_In and from 9 to 10 for Fe_2_P → CaC_2_. This is unlike those transitions of unchanged CN (e. g., 9 to 9 in OII → Fe_2_P), for which a very slight change in enthalpy has been reported in TiO_2_, ZrO_2_ and HfO_2_^23,35,53^.

**Table 3:** Enthalpy difference and its components with respect to pressure and volume change across Fe_2_P → Ni_2_In, Fe_2_P → CaC_2_ and Fe_2_P → AlB_2_ phase transitions in ZrO_2_.

Phase transition	Δ*H*/Δ*P*		Δ*E*/Δ*P*		Δ(*PV*)/Δ*P*		Volume change (%)
	eV GPa^-1^ (× 10^–4^)	kJ mol^-1^ GPa^-1^	eV GPa^-1^ (× 10^–4^)	kJ.mol^-1^ GPa^-1^	eV GPa^-1^ (× 10^–4^)	kJ mol^-1^ GPa^-1^	
Fe_2_P → Ni_2_In	− 57.787	− 0.55755	+ 7.187	+ 0.06935	− 64.974	− 0.62690	4.7
Fe_2_P → CaC_2_	− 41.511	− 0.40052	+ 0.057	+ 0.00055	− 41.568	− 0.40107	4.1
Fe_2_P → AlB_2_	− 45.035	− 0.43452	+ 0.035	+ 0.00034	− 45.070	− 0.43486	5.1

Additionally, we should note that although the transition to Ni_2_In is associated with a CN increase of 2 (11-fold) and the transition to CaC_2_ is associated with a CN increase of 1(tenfold), the volume change across both transitions is similar (Table 3). In fact, it has been obviously shown that for ZrO_2_ and other similar dioxides, the 2-CN increase (from 7 to 9 in OI → OII transition) results in ~ 10% volume collapse^25,39^. Thus, we conclude that a volume reduction of 4.7% in the Fe_2_P → Ni_2_In is too low for 2-CN increase in this transition, while the 4.1% reduction in Fe_2_P → CaC_2_ transition is more reasonable for 1-CN increase. Moreover, we should note that the volume reduction across Fe_2_P → CaC_2_ transition in TiO_2_ is found to be 3.3–3.4%^41,42^, which agrees well with our result (4.1%) for the ZrO_2_ case. Consequently, in terms of the correlation between the volume drop and the CN change, the transition from Fe_2_P phase to CaC_2_ phase is more likely to happen in zirconia compared to Ni_2_In phase.

### Band gap calculations across phase transitions

**Figure 5:**
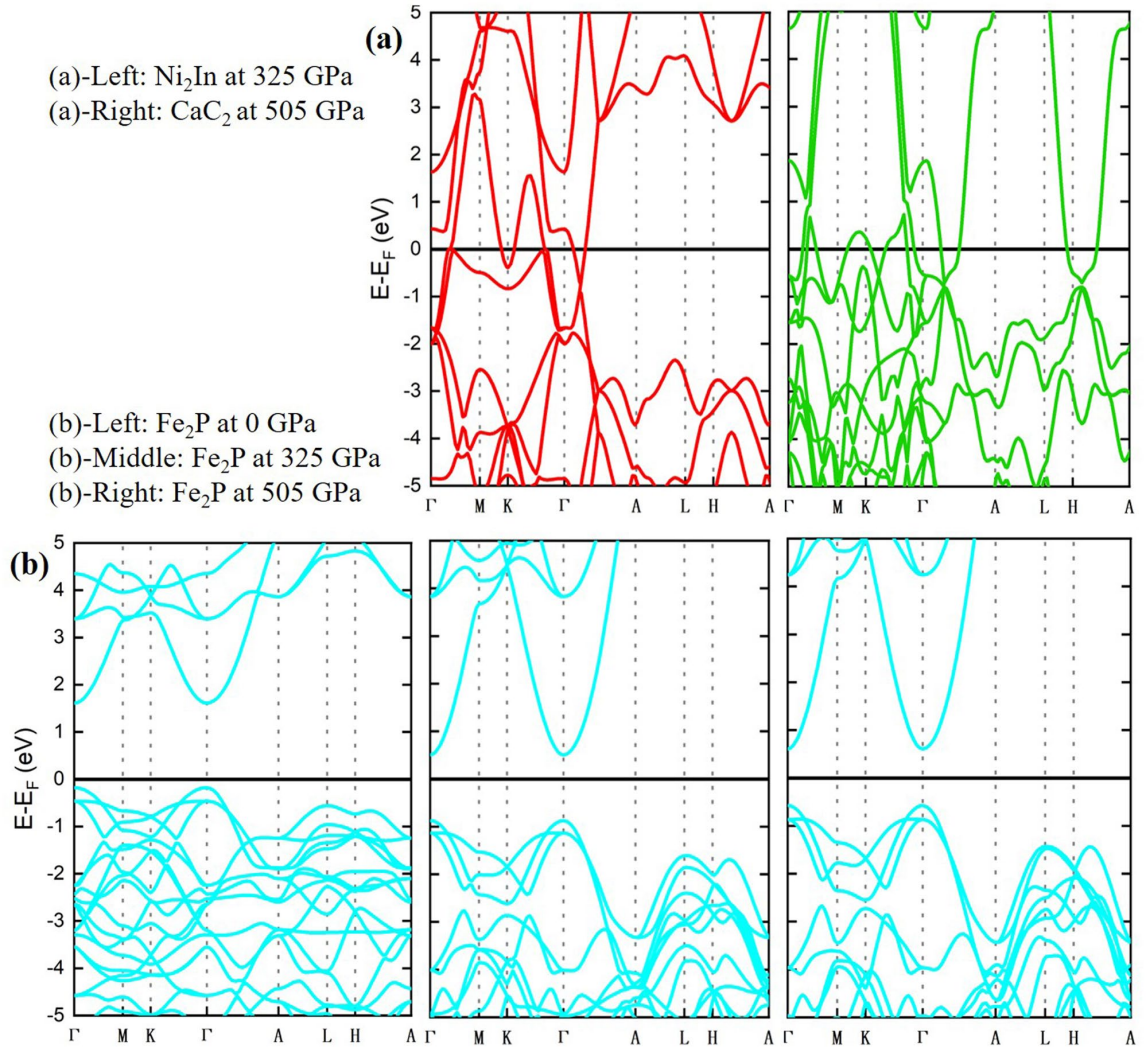
Electronic band structure of: (**a**) Ni_2_In and CaC_2_ phases at 325 GPa and 505 GPa, respectively. (**b**) Fe_2_P phase at 0 GPa, 325 GPa, and 505 GPa.

To get a deeper insight into the phase transitions of ZrO_2_ at ultrahigh pressures, and to obtain a better prediction of the favored transition from Fe_2_P phase, we have performed band gap calculations to study the effect of pressure on the band gap of Fe_2_P, Ni_2_In and CaC_2_ phases. Interestingly, our calculations reveal that both Ni_2_In and CaC_2_ phases are metallic (zero band gap) from 0 GPa up to their transition pressures from Fe_2_P phase: 325 GPa for Ni_2_In and 505 GPa for CaC_2_ (Fig. 5a). On the other hand, the calculated band gap of Fe_2_P at zero pressure is found to be 1.78 eV, and it decreases to 1.38 eV and 1.18 eV at 325 GPa and 505 GPa, respectively (Fig. 5b). However, regardless of such narrowing in the band gap at high pressures, Fe_2_P retains its semiconducting characteristic. Even though Fe_2_P does not change from semiconductor to metallic up to 505 GPa, the band gap decrease with increasing pressure likely reveals an evidence for metallization of Fe_2_P at higher pressures, in agreement with previous findings on TiO_2_^41,42^. Obviously, we note that the band gap difference across Fe_2_P → CaC_2_ transition (1.18 eV → 0 eV) is less than the difference across Fe_2_P → Ni_2_In transition (1.38 eV → 0 eV). Therefore, our findings from the band gap point of view, indicate that the Fe_2_P → CaC_2_ transition is likely favored in comparison to the Fe_2_P → Ni_2_In transition.

## Conclusions

In summary, we have used DFT calculations to study the phase relations among various structures of ZrO_2_ in the ultrahigh pressure regime. We found that Ni_2_In-type structure is neither stable with respect to CaC_2_-type structure nor to AlB_2_-type structure. A special focus has been made to investigate the Fe_2_P → Ni_2_In and Fe_2_P → CaC_2_ transitions for the sake of determining the favored transition from Fe_2_P-ZrO_2_. Our analysis across these transitions, in terms of the volume reduction, the CN increase, and the band gap narrowing, demonstrates that Fe_2_P → CaC_2_ is more likely to occur in zirconia at megabar pressures. Therefore, our calculations predict CaC_2_-type structure to be likely the post-Fe_2_P phase at ultrahigh pressure when compared to Ni_2_In-type structure, which is also evidenced by a similar prediction previously obtained in the transition-metal dioxide TiO_2_.

## Data Availability

The data presented in this study are available in the article; however, more details regarding the data will be available upon reasonable request.
